# First freshwater mussel-associated piscicolid leech from East Asia

**DOI:** 10.1038/s41598-020-76854-0

**Published:** 2020-11-16

**Authors:** Ivan N. Bolotov, Anna L. Klass, Ekaterina S. Konopleva, Yulia V. Bespalaya, Mikhail Yu. Gofarov, Alexander V. Kondakov, Ilya V. Vikhrev

**Affiliations:** 1 Northern Arctic Federal University, Arkhangelsk, Russia; 2 N. Laverov Federal Center for Integrated Arctic Research of the Ural Branch of the Russian Academy of Sciences, Arkhangelsk, Russia

**Keywords:** Zoology, Phylogenetics, Taxonomy

## Abstract

Parasites and symbionts of freshwater mussels are poorly understood, although a diverse assemblage of mussel-associated leeches (Glossiphoniidae) was recently described. Here, we report on the discovery of a fish leech (Piscicolidae) in the mantle cavity of the freshwater mussel *Cristaria plicata* (Unionidae) in the Russian Far East. It is the first member of this leech family being associated with freshwater molluscs. This leech does not match any known genus and species both morphologically and genetically, and is described here as *Alexandrobdella makhrovi* **gen. & sp. nov**. It uses mussels as shelter (and probably as a secondary host), while the Amur catfish *Silurus asotus* (Siluridae) seems to be the primary host. These novel findings indicate that mussel-associated leech assemblage contains at least one piscicolid species. Our fossil-calibrated phylogeny suggests that the crown group of Piscicolidae was originated in the Early Cretaceous. This primarily marine family shares at least five independent colonization events into freshwater environments.

## Introduction

Mussel-associated leeches are a species-rich ecological group, examples of which have been described from North America, Africa, India and Nepal, Southeast Asia, and East Asia^1,2^. It was shown that most of mussel-associated leeches use freshwater mussels (order Unionida) as secondary hosts and shelter, while various freshwater fish species serve as the primary host^2^. All mussel-associated leeches known to date belong to three genera in the family Glossiphoniidae, i.e., *Batracobdelloides* Oosthuizen, 1986, *Hemiclepsis* Vejdovsky, 1884, and *Placobdella* Blanchard, 1893^2^. Furthermore, several glossiphoniid leeches are associated with freshwater gastropods, with examples reported from Africa^3^, North America^4^, and Southeast Asia^5^. The freshwater leech *Alboglossiphonia polypompholyx* Oosthuizen, Hussein & El-Shimy, 1988 from Egypt seems to be a unique example of an obligate parasite of the mantle cavity of freshwater snails^3,6^, although a number of taxa could be overlooked due to the hidden life style of such small leeches.

None of the fish leeches (Piscicolidae) was known to occur in association with freshwater mollusks, although the marine fish leech *Pontobdella moorei* (Oka, 1910) uses *Octopus bimaculatus* Verrill, 1883 (Cephalopoda: Octopodidae) as the primary host^7^. This leech family contains numerous marine taxa (including several species discovered from oceanic trenches up to 8.7 km deep^8^) alongside with a few radiations in fresh water^9–11^. However, the origin of fresh- and brackish-water lineages of Piscicolidae is still unclear. Lukin^12^ and Epshtein^13^ hypothesized that freshwater piscicolid leeches originated from marine ancestors. Later, this hypothesis was corroborated based on molecular data obtained from mitochondrial DNA^14^. It is currently accepted that the family Piscicolidae contains three subfamilies: Piscicolinae Johnston, 1865, Platybdellinae Epshtein, 1970, and Pontobdellinae Llewellyn, 1966^14,15^. The first subfamily was recovered as non-monophyletic using a DNA-based approach^16^. Recently, the family Piscicolidae together with the Ozobranchidae were placed in the separate suborder Oceanobdelliformes^17^.

This correspondence (1) reports on the first example of an association of a piscicolid leech species (Hirudinea: Piscicolidae) with freshwater mussels; (2) describes this leech as a genus and species new to science; (3) reconstructs a time-calibrated phylogeny of the piscicolid leeches, and (4) discusses the origin of freshwater lineages in this family in a broader evolutionary context.

## Results

**Figure 1:**
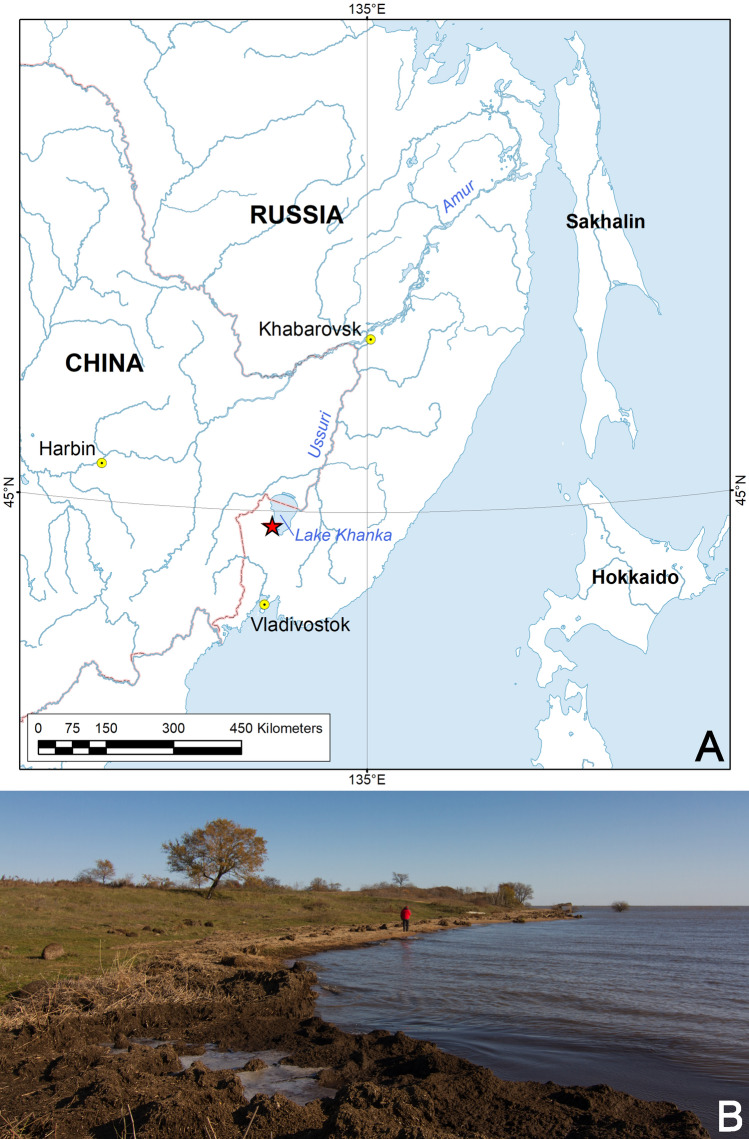
Type locality of *Alexandrobdella makhrovi* **gen. & sp. nov.** (**A**) Geographic position of the type locality (red star). The map was created using ESRI ArcGIS 10 software (https://www.esri.com/arcgis); the topographic base of the map was created with free open sources such as Natural Earth Free Vector and Raster Map Data (https://www.naturalearthdata.com), Global Self-consistent Hierarchical High-resolution Geography, GSHHG v2.3.7 (https://www.soest.hawaii.edu/wessel/gshhg), and HydroSHEDS (https://www.hydrosheds.org). (**B**) Coastal area of Lake Khanka at the type locality. Photo: Ilya V. Vikhrev.

**Figure 2:**
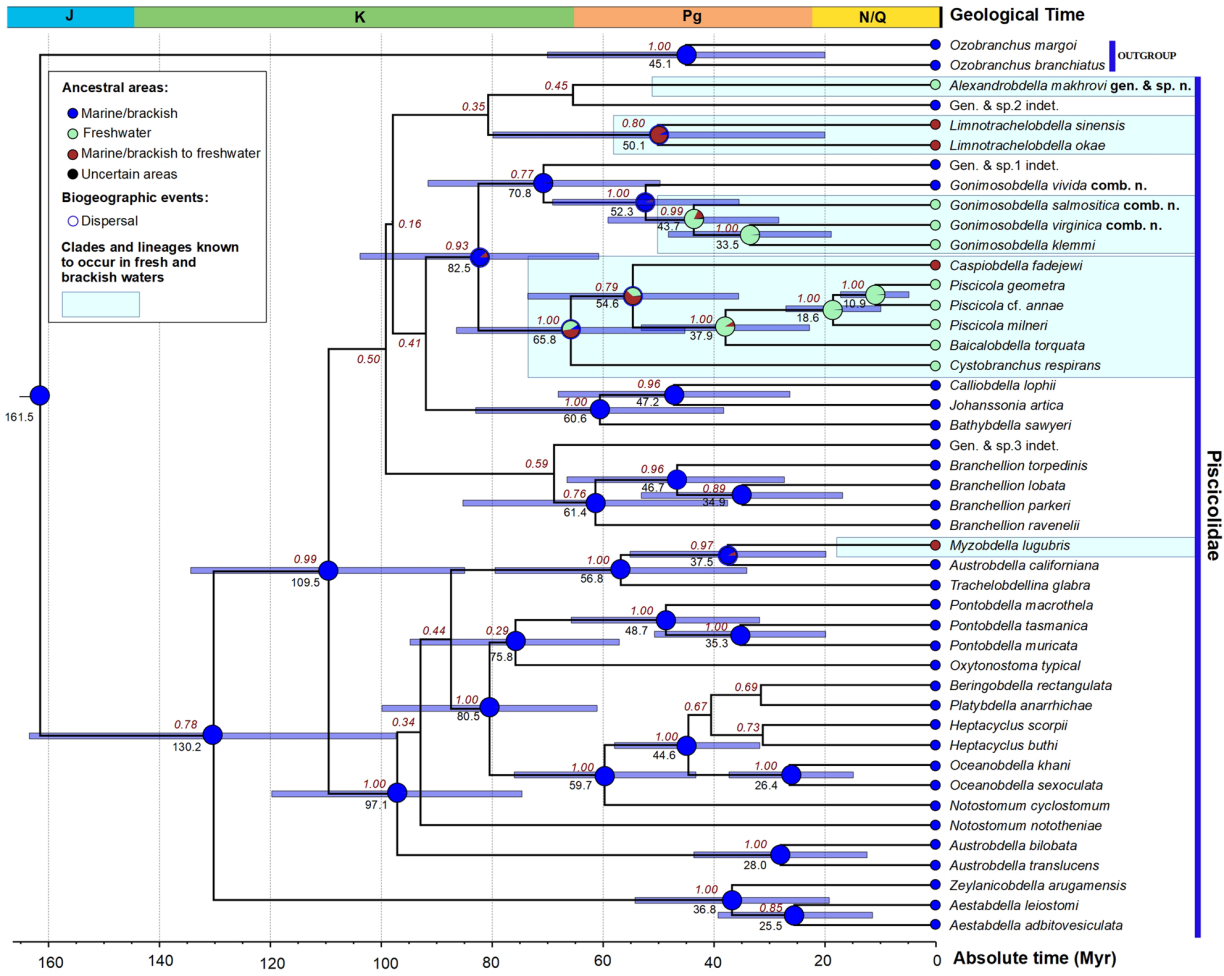
Bayesian time-calibrated phylogeny (four partitions: three codons of COI + 18S rRNA) and ancestral environment reconstruction of the Piscicolidae. Brown numbers near nodes are BPP values of BEAST. Black numbers near nodes are node ages (Myr). The tip circles indicate environmental preference of leech species: freshwater (blue); marine (green); and euryhaline (brown) (Table 1). Reconstructions for weakly supported nodes (BPP < 0.75) are omitted. Two Ozobranchidae taxa were used as outgroup.

**Figure 3:**
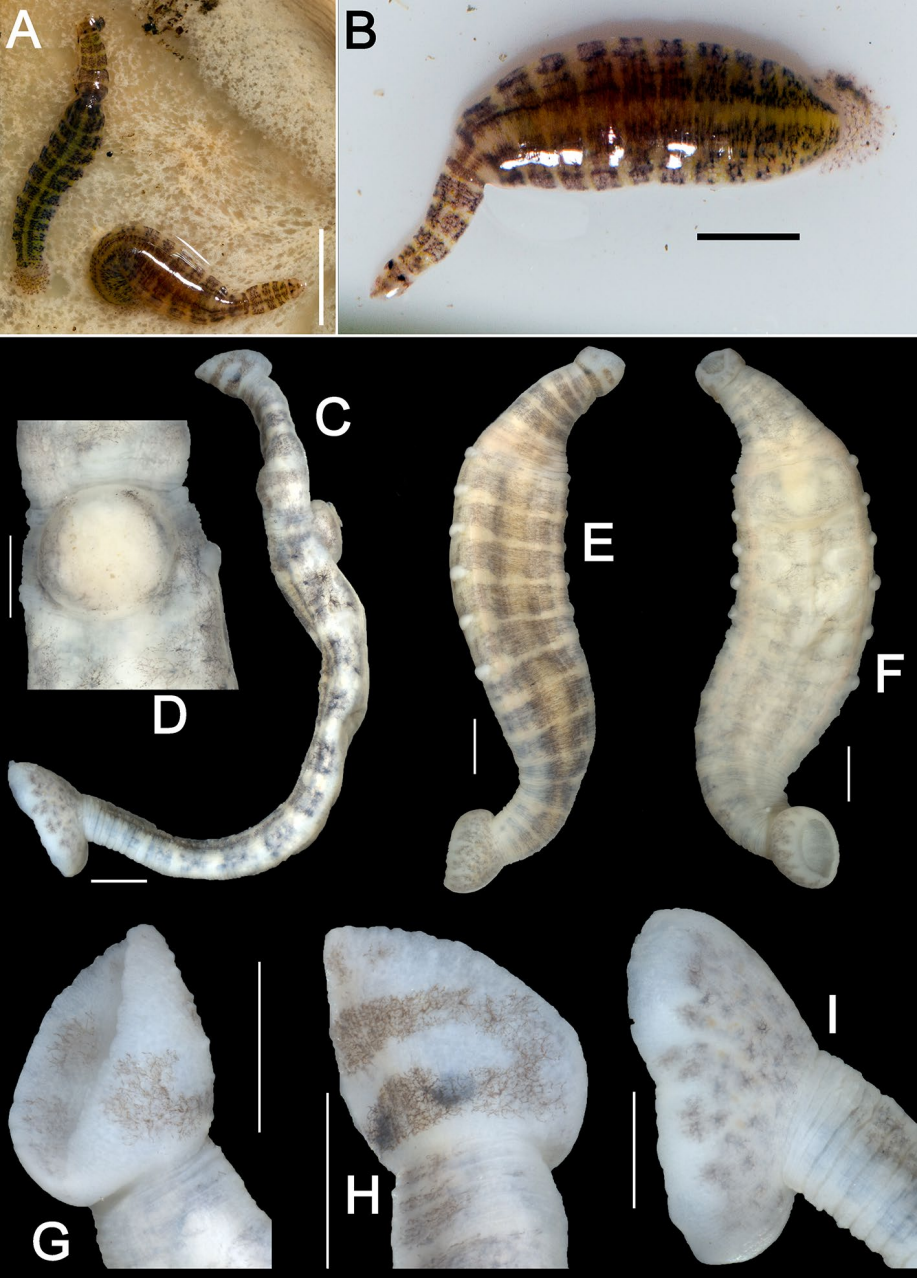
Morphology of the type specimens of *Alexandrobdella makhrovi* **gen. & sp. nov.** (**A**) Living holotype Hir_0084/2-H and paratype RMBH Hir_0084/1-P specimens (dorsal view) in the mantle cavity of their host mussel *Cristaria plicata* (Bivalvia: Unionidae), 24.v.2017. (**B**) Living paratype Hir_0084/1-P (dorsal view), 24.v.2017. (**C**) Holotype Hir_0084/2-H (lateral view). (**D**) Clitellum of the holotype (ventral view). (**E**) Paratype Hir_0086-P (dorsal view). (**F**) Paratype Hir_0086-P (ventral view). (**G**) Anterior sucker of the holotype (lateral view). (**H**) Anterior sucker of the holotype (dorsal view). (**I**) Caudal sucker of the holotype (lateral view). Scale bars = 5 mm [A], 2 mm [B], and 1 mm [C-I]. Photos: Ilya V. Vikhrev [A-B] and Anna L. Klass [C-I].

**Figure 4:**
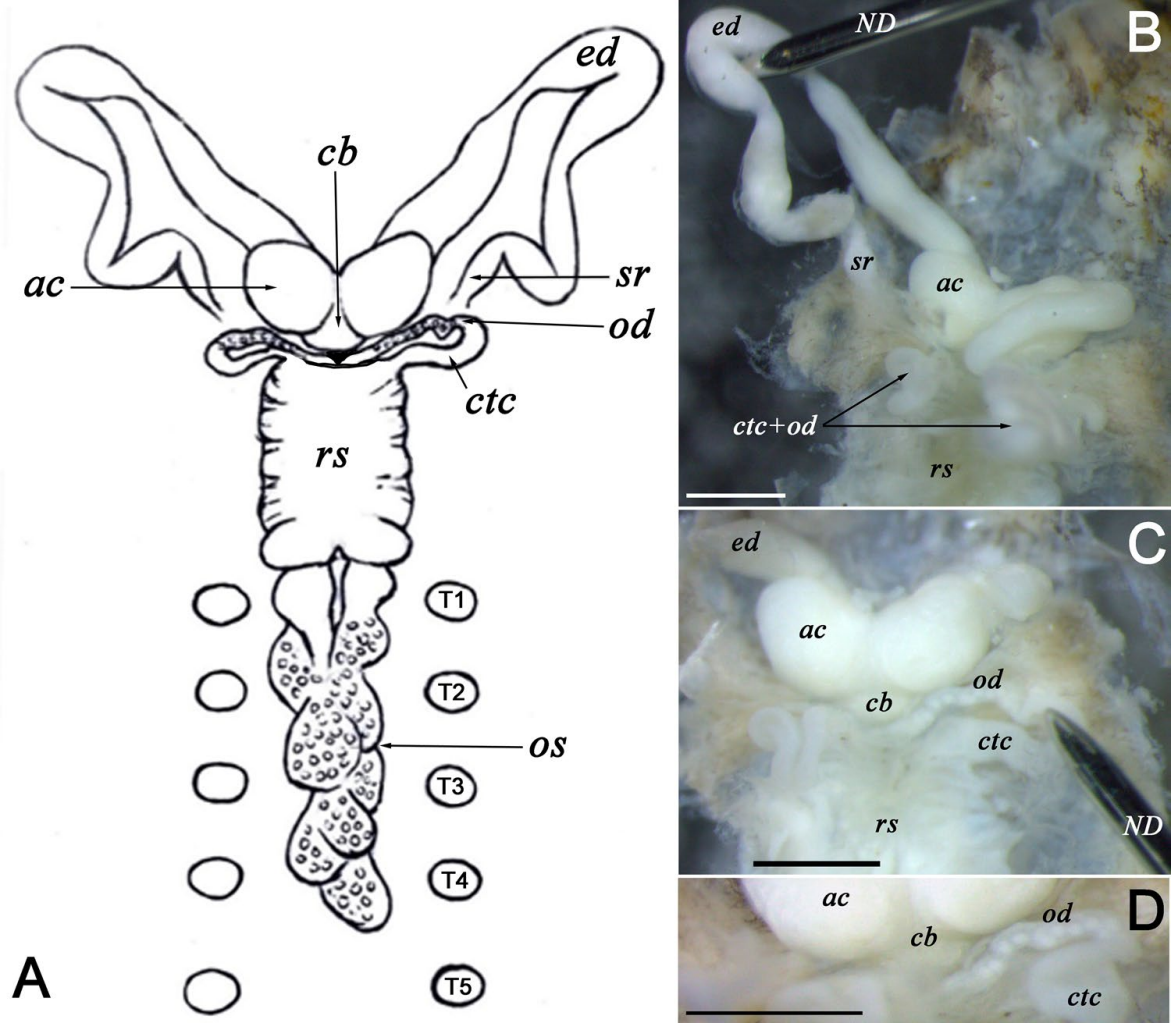
Reproductive system of *Alexandrobdella makhrovi* **gen. & sp. nov.** (dorsal view). (**A**) General scheme of the system (graphics). (**B**) Left ejaculatory duct and seminal reservoir expanded. (**C**) Right oviduct and conductive tissue cord expanded. (**D**) Same characters but with higher magnification. Abbreviations: *ac* atrial cornu (terminal parts of the ejaculatory ducts); *cb* copulatory bursa; *ed* ejaculatory ducts; *sr* seminal reservoir; *ctc* conductive tissue cords; *rs* seminal receptacle; *os* ovisacs; *od* oviducts; *T* testisac with corresponding number; and *ND* dissecting needle. Scale bars = 0.5 mm. Graphics and photos: Anna L. Klass.

A sample of fish leeches collected from the mantle cavity of the freshwater mussel species *Cristaria plicata* (Bivalvia: Unionidae) from Lake Khanka (Fig. 1, Tables 1, 2) shares a distinctive set of morphological and molecular characters and is described here as *Alexandrobdella makhrovi* **gen. & sp. nov.** (Figs. 2, 3, 4). Our DNA analyses indicate that the crop of a paratype of *Alexandrobdella makhrovi* **gen. & sp. nov.**[RMBH Hir_0084/1-P] was filled by blood of the Amur catfish *Silurus asotus* Linnaeus, 1758 (Siluridae) [GenBank accession no. MT707651]. Therefore, freshwater fish can be considered the primary hosts of *Alexandrobdella*, while the nature of its association with freshwater mussels (i.e., shelter only or a secondary host as well) remains largely unclear.

**Table 1:** List of *COI* and *18S rRNA* gene sequences of the Hirudinea used in this study.

Genus	Species	*COI*	*18S rRNA*	Haplotype Code	Region	Environment*
**In-group taxa**						
**Piscicolidae**						
*Alexandrobdella* **gen. nov**	*A. makhrovi* **gen. & sp. nov**	**MN295413**	**MN312187**	AleMak	East Asia	Freshwater
*Limnotrachelobdella* Epshtein, 1968	*L. sinensis* (Blanchard, 1896)	LC275140	LC275139	LimSin	East Asia	Brackish to freshwater
	*L. okae* (Moore, 1924)	AY336022	N/A	LimOka	East Asia	Marine to freshwater
Gen.1 indet	Gen. & sp. indet. HLC-30322	MG421319	N/A	PisSp1	North America	Marine
Gen.2 indet	Gen. & sp. indet. PT-2003	AY336023	N/A	PisSp2	East Asia	Brackish to marine
Gen.3 indet	Gen. & sp. indet. Aio2018LYKM	LC460256	N/A	PisSp3	East Asia	Marine
*Caspiobdella* Epshtein, 1966	*C. fadejewi* (Epshtein, 1961)	AY336020	N/A	CasFad	Europe	Brackish to freshwater
*Piscicola* Blainville, 1818	*P. geometra* (Linnaeus, 1761)	AY336014	AF099946	PisGeo	Europe	Freshwater
	*P. milneri* (Verrill, 1874)	DQ414337	DQ414292	PisMil	North America	Freshwater
	*P.* cf. *annae* Bielecki, 1997	AY336016	N/A	PisAnn	Europe	Freshwater
*Branchellion* Savigny, 1822	*B. torpedinis* Savigny, 1822	AF003265	AF115993	BraTor	North America	Marine
	*B. lobata* Moore, 1952	DQ414307	DQ414261	BraLob	North America	Marine
	*B. parkeri* Richardson, 1949	DQ414308	DQ414262	BraPar	Australia	Marine
	*B. ravenelii* (Girard, 1851)	DQ414309	DQ414263	BraRav	Central America	Marine
*Calliobdella* van Beneden & Hesse, 1863	*C. lophii* Van Beneden & Hesse, 1863	DQ414314	DQ414268	CalLop	Europe	Marine
*Myzobdella* Leidy, 1851	*M. lugubris* Leidy, 1851	AF003269	AF115994	MyzLug	North America	Marine to freshwater
*Pontobdella* Leach, 1815	*P. macrothela* (Schmarda, 1861)	AF116022	AF115996	PonMac	North America	Marine
	*P. tasmanica* (Hickman, 1942)	DQ414343	DQ414298	PonTas	Australia	Marine
	*P. muricata* (Linnaeus, 1758)	KY659072	KY659070	PonMur	Europe	Marine
*Zeylanicobdella* Silva, 1963	*Z. arugamensis* de Silva, 1963	DQ414344	DQ414299	ZeyAru	Southeast Asia	Marine
*Aestabdella* Burreson, 1976	*A. leiostomi* Burreson, 1991	DQ414305	DQ414259	AesLei	North America	Marine
	*A. adbitovesiculata* (Moore, 1952)	DQ414300	DQ414254	AesAbd	Hawaii	Marine
*Cystobranchus* Diesing, 1859	*C. respirans* (Troschel, 1850)	AY336021	N/A	CysRes	Europe	Freshwater
*Baicalobdella* Dogiel & Bogolepova, 1957	*B. torquata* Grube, 1871	AY336018	N/A	BaiTor	East Asia	Freshwater
*Gonimosobdella* Williams & Burreson, 2005	*G. klemmi* Williams & Burreson, 2005	DQ414318	DQ414272	GonKle	North America	Freshwater
	*G. salmositica* (Meyer, 1946) **comb. nov**	DQ414316	DQ414270	CysSal	North America	Freshwater
	*G. virginica* (Hoffman, 1964) **comb. nov**	DQ414317	DQ414271	CysVir	North America	Freshwater
	*G. vivida* (Verrill, 1872) **comb. nov**	AF003260	AF115992	CalViv	North America	Marine
*Bathybdella* Burreson, 1981	*B. sawyeri* Burreson, 1981	DQ414311	DQ414265	BatSaw	East Pacific Rise	Marine
*Johanssonia* Selensky, 1914	*J. artica* (Johansson, 1898)	DQ414320	DQ414274	JohArc	North America	Marine
*Austrobdella* Badham, 1916	*A. bilobata* Ingram, 1957	DQ414301	DQ414255	AusBil	Australia	Marine
	*A. translucens* Badham, 1916	DQ414306	DQ414260	AusTra	Australia	Marine
	*A. californiana* Burreson, 1977	DQ414304	DQ414258	AusCal	North America	Marine
*Beringobdella* Caballero, 1974	*B. rectangulata* (Levinsen, 1881)	DQ414310	DQ414264	BerRec	East Asia	Marine
*Platybdella* Malm, 1863	*P. anarrhichae* (Diesing, 1859)	DQ414336	DQ414291	PlaAna	Europe	Marine
*Heptacyclus* Vasileyev, 1939	*H. scorpii* (Malm, 1863)	DQ414326	DQ414280	MalSco	North America	Marine
	*H. buthi* (Burreson & Kalman, 2006)	DQ414322	DQ414276	MalBut	North America	Marine
*Oceanobdella* Caballero, 1956	*O. khani* Burreson & Williams, 2004	DQ414331	DQ414286	OceKha	East Asia	Marine
	*O. sexoculata* (Malm, 1863)	DQ414332	DQ414287	OceSex	North America	Marine
*Notostomum* Levinsen, 1882	*N. cyclostomum* Johansson, 1898	DQ414327	DQ414282	NotCyc	East Asia	Marine
*Notobdella* Benham, 1909	*N. nototheniae* Benham, 1909	DQ414330	DQ414285	NotNot	Antarctica	Marine
*Oxytonostoma* Malm, 1863	*O. typical* Malm, 1863	DQ414333	DQ414288	OxyTyp	North America	Marine
*Trachelobdellina* Moore, 1957	*T. glabra* Moore, 1957	EF405597	N/A	TraGla	Antarctica	Marine
**Outgroup taxa**						
**Ozobranchidae**						
*Ozobranchus* Quatrefages, 1852	*O. branchiatus* (Menzies, 1791)	KF728213	KF728214	OzoBra	North America	Marine
	*O. margoi* (Apáthy, 1890)	KJ451407	KF728217	OzoMar	North America	Marine

*N/A* not available. *Data on environmental preferences was obtained from the IRMNG database (https://www.irmng.org^33^ and published sources^19,34^.

**Table 2:** Voucher numbers, reference DNA sequences, and measurements of the type series of *Alexandrobdella makhrovi* **gen. & sp. nov.**

Status of specimen	Voucher no.*	*COI* acc. no	*18S rRNA* acc. no	Measurements (mm)**			
				BL	BW	AW	PW
Holotype	RMBH Hir_0084/2-H	N/A	N/A	14.5	2.20	1.10	1.95
Paratype	RMBH Hir_0084/1-P	MN295413	MN312187	12.6	3.36	1.03	1.75
Paratype	RMBH Hir_0086-P	N/A	N/A	11.0	2.26	0.84	1.41

*N/A* not available. *Type series is deposited in the *RMBH* Russian Museum of Biodiversity Hotspots, Federal Center for Integrated Arctic Research of the Ural Branch of the Russian Academy of Sciences, Arkhangelsk, Russia. **Measurements of leech specimens (mm): *BL* body length, *BW* body width, *AW* width of anterior sucker, and *PW* width of posterior sucker.

The deep subfamily-level nodes of our two-locus Bayesian phylogeny (*COI* + *18S rRNA*) were rather weakly supported, while shallower nodes corresponding to generic and intra-generic clades shared high and moderate support values (BPP ≥ 0.95 and ≥ 0.75, respectively). Phylogenetically, *Alexandrobdella makhrovi* **gen. & sp. nov.** was recovered within a weakly supported clade that contains *Limnotrachelobdella* spp. and an unidentified marine leech species (Fig. 2). This group appears to be a part of the subfamily Piscicolinae, which was also weakly supported in our research.

Our ancestral trait modeling suggested that there were at least five independent colonization events of piscicolids to fresh and brackish water: (i) *Piscicola* + *Baicalobdella* + *Cystobranchus respirans* + *Caspiobdella* (BPP = 1.00); (ii) *Gonimosobdella klemmi* + *G. salmositica* **comb. nov.** + *G. virginica* **comb. nov.** (BPP = 0.99); (iii) *Limnotrachelobdella* (BPP = 0.80); (iv) *Myzobdella lugubris* (BPP = 0.97); and (v) *Alexandrobdella makhrovi* **gen. & sp. nov.** (BPP = 0.45) (Fig. 2).

### Taxonomy

Family Piscicolidae Johnston, 1865.

Genus *Alexandrobdella* **gen. nov.**

**LSID:** https://zoobank.org/urn:lsid:zoobank.org:act:E644FB76-B5F2-4405-8D13-982D362E790C.

**Type species:** *Alexandrobdella makhrovi* **gen. & sp. nov.**

**Etymology.** This genus is named for Dr. Alexander Makhrov, a prominent Russian ichthyologist and evolutionary biologist, and “bdella”, the Greek word for leech.

**Diagnosis.** Body flattened dorso-ventrally, clearly divided into short trachelosome and elongated urosome (Fig. 3). Both trachelosome and urosome taper towards corresponding suckers. Suckers well developed. Pulsatile vesicles present, rather large, knob-like. One pair of large eyes on oral sucker. Segmental and caudal ocelli absent. Urosomal segments 7(14) annulate. Five pairs of testisacs. Large, rectangular muscular organ (seminal receptacle) associated with bursa and ovisacs. Conductive tissue present. Conductive tissue cords conduct oviducts with the seminal receptacle (Fig. 4). Ovisacs massive, asymmetric, lobed. There are no freshwater genera of the Piscicolidae having such combination of morphological characters, although *Limnotrachelobdella* Epshtein, 1968 and *Taimenobdella* Epshtein, 1987 appear to be more closely related to the new genus externally. However, *Alexandrobdella* **gen. nov.** differs from *Limnotrachelobdella* Epshtein, 1968 by having smaller, knob-like pulsatile vesicles, a less distinct separation of the body into trachelosome and urosome, and a smaller posterior sucker. It can be distinguished from *Taimenobdella* Epshtein, 1987 by having one pair of eyes and larger pulsatile vesicles, and by the lack of segmental and caudal ocelli. Based on the reproductive system morphology (Fig. 4), the new genus can be distinguished from all the other Piscicolidae genera by having an exceptionally large seminal receptacle and massive, asymmetrical, lobed ovisacs. The Antarctic genus *Trulliobdella* Brinkmann, 1948 is partly similar to the new genus in that it shares oviducts connecting with the seminal receptacle by conductive tissue cords^18^. However, *Alexandrobdella* **gen. nov.** has a much larger, rectangular seminal receptacle and different morphology of ovisacs compared with those in *Trulliobdella* species.

**Phylogenetic placement.** It is clear that *Alexandrobdella* **gen. nov.** represents a highly divergent lineage, which is distant phylogenetically from other freshwater and marine piscicolid genera, the DNA sequences of which were available (Fig. 2 and Table 1). This genus appears to be more closely related to *Limnotrachelobdella* and an unidentified marine leech Gen.2 indet. but these relationships were poorly supported in our phylogeny (BPP = 0.35–0.45).

*Alexandrobdella makhrovi* **gen. & sp. nov.**

Figures 1, 2, 3, 4; Table 2.

**LSID:** https://zoobank.org/urn:lsid:zoobank.org:act:2723D77F-9AC1-4E41-AA21-1952FD97A94B.

**Common name:** Alexander Makhrov Leech.

**Holotype** RMBH **Hir_0084/2-H**, RUSSIA: Lake Khanka, 44.7065°N, 132.0728°E, 24.v.2017, Bolotov, Makhrov, and Vikhrev leg.

**Paratypes.** RUSSIA: type locality, same collectors, 24.v.2017, one sequenced specimen [RMBH **Hir_0084/1-P**; Table 2], 25.v.2017, one specimen [RMBH **Hir_0086-P**].

**Mussel host.** The type series was collected from the mantle cavity of the freshwater mussel species *Cristaria plicata* (Leach, 1814) (Bivalvia: Unionidae).

**Fish host.** The Amur catfish *Silurus asotus* Linnaeus, 1758 (Siluridae) [GenBank accession no. MT707651]. The host range of this leech needs future studies as the crop content of only one leech was sequenced.

**Etymology.** This novel species is dedicated to our friend and colleague Dr. Alexander Makhrov as is the new genus. Alexander helped us to collect the type series from Lake Khanka.

**Morphological diagnosis.** *External morphology:* Small leech, body flattened dorso-ventrally, clearly divided into trachelosome and urosome. Body length with suckers up to 14.5 mm, width up to 3.4 mm (at the widest part of the urosome) (Table 2). Skin smooth, without papillae, 11 pairs of knob-like pulsatile vesicles laterally (especially noticeable during pregnancy). Integuments weakly pigmented, light yellow in fixed specimens (translucent in living leeches) with a black and dark brown pigmentation. On dorsal side accumulations of pigment form dark brown transverse stripes, which together with unpigmented areas form a characteristic ‘mosaic’ pattern. Ventral side light yellow, almost unpigmented. Anterior sucker small, distinctly separated, approximately as broad as the width of trachelosome, eccentrically attached. One pair of eyes located on a brown stripe near the border with trachelosome. Posterior sucker medium sized (diameter approximately 1.5–2 times larger than that of the anterior sucker), eccentrically attached, eye-like spots absent, but yellow–orange spots present. Complete somite contains seven double rings. Anus separated from the posterior sucker by two annuli. *Digestive system:* Proboscis medium long, muscular. Oesophagus surrounded by fine salivary glands. The number of chambers of the crop in the sample was not determined. Posterior crop caeca fused incompletely, persist five fenestrae. Intestine with four chambers bearing lateral processes. Rectal dilatation located posteriorly to the posterior crop caeca. *Reproductive system:* Gonopores are separated by two annuli. Testisacs 5 pairs, relatively large, oval. Seminal reservoirs short. Ejaculatory ducts long, muscular, forming loops. Terminal parts of the ejaculatory ducts voluminous, spherical. Copulatory bursa small. Accessory glands not found. Ovisacs long, massive, consisting of some lobes, asymmetrical, located posteriorly of the voluminous seminal receptacle. Oviducts connected by conductive tissue cords with the seminal receptacle, which forms internal copulatory area. Externally, the copulatory area could appear as swelling of the ventral surface of clitellum.

**Life style.** At first glance, this new leech appears to be a mussel-associated species that uses the mantle cavity of a freshwater mussel as shelter. However, its relationship with freshwater mussels (i.e., shelter only or a host-parasite association) requires further research. The proportion of *Cristaria plicata* mussels infested by at least one leech in our sample from the type locality was 9.7%, and the intensity of leech infestation there was 0.10 ± 0.05 leeches per mussel (mean ± s.e.m.; *N* = 31 mussels and 3 leeches).

**Distribution.** This species is known only from its type locality in the southwestern part of Lake Khanka, Russian Far East (Fig. 1).

## Discussion

### First association of a piscicolid leech with freshwater mussels

Earlier research revealed that global mussel-associated leech assemblage includes at least 12 species belonging to the family Glossiphoniidae^2^. Our novel discovery of a piscicolid leech in the mantle cavity of a freshwater mussel from East Asia expands our knowledge of mussel-leech associations. The DNA analyses of the crop content of an *Alexandrobdella makhrovi* **gen. & sp. nov.** paratype indicated that the adult leech feeds on fish blood, i.e., uses the Amur catfish (and probably other freshwater fish species as well) as the primary host. However, nothing is known on the interactions between this leech and its host mussel. The mussel-associated glossiphoniids (*Hemiclepsis* and *Batracobdelloides*) use freshwater mussels as shelters and secondary hosts but adult leeches need to take one or several fish-blood meals to complete their life cycle^2^.

Freshwater piscicolid leeches are known to feed on fish blood almost exclusively^9,19^, while one species (*Gonimosobdella virginica* **comb. nov.**) was found to be a possible obligate egg feeder in nests of four fish taxa^20^. Conversely, the marine Piscicolidae share a much broader host range, with numerous species being associated with vertebrate and invertebrate taxa such as fishes, turtles, crustaceans, pycnogonids, and octopuses^7,21–24^. A brief review of the body of literature, outlined above, revealed that piscicolid leeches could use a variety of invertebrate animals as hosts, at least in marine environments. Hence, it is unclear whether *Alexandrobdella* feeds on its mussel host or uses it as available shelter only. Possible host–parasite relationships of the fish leech with freshwater mussels deserve further research efforts.

### Taxonomic issues

A new genus and species, *Alexandrobdella makhrovi* **gen. & sp. nov.**, are introduced here for a mussel-associated fish leech from East Asia. This leech clearly differs from other members of the family by having an enormous seminal receptacle, which posteriorly connects with large, asymmetrical, lobed ovisacs. In its turn, the proximal parts of oviducts connected with the seminal receptacle by conductive tissue cords.

Based on our two-locus phylogeny, two freshwater *Cystobranchus* and one marine *Calliobdella* species from North America are transferred here to *Gonimosobdella* (Table 1), as it was already suggested in a dissertation^19^. The new combinations are proposed as follows: *Gonimosobdella salmositica* (Meyer, 1946) **comb. nov.**, *G. virginica* (Hoffman, 1964) **comb. nov.**, and *G. vivida* (Verrill, 1872) **comb. nov.**

### Multiple colonization events of marine fish leeches into fresh water

Our ancestral trait modeling supports the hypothesis that the Piscicolidae is primarily a marine group of leeches^14,17^. The crown group of this clade was likely originated in the Early Cretaceous. There were at least five independent colonization events of the marine fish leeches to fresh and brackish water environments since the Late Cretaceous. Multiple independent colonization events of piscicolids to oceanic trenches were also recorded, with subsequent morphological and ecological adaptations to extreme deep-sea environments^8,25^.

## Methods

### Data sampling and molecular analyses

Leeches were collected from the mantle cavity of living freshwater mussels *Cristaria plicata* (Bivalvia: Unionidae) that were found near the shore of Lake Khanka after a strong storm. Their habitat was a shallow littoral area of the lake with silty–sand bottom (Fig. 1). The mussel specimens were opened with an extractor and their mantle cavity was visually examined for leeches and other inhabitants that were sampled by forceps^2^. During the 2-day period (24–25.v.2017), we examined 31 specimens of *Cristaria plicata*, and collected three individuals of *Alexandrobdella makhrovi* **gen. & sp. nov**. The leech infestation prevalence (%) and intensity of leech parasitism estimates were calculated using the equations described in our previous papers^1,2^.

We obtained sequences of the mitochondrial *cytochrome c oxidase subunit I* (*COI*) and the nuclear *small subunit of ribosomal RNA* (*18S rRNA*) gene fragments from one specimen of the novel species (paratype RMBH Hir_0084) using the laboratory protocols and primers followed those described in our earlier work^2^.

### Divergence time estimates and statistical biogeography

We sampled a comprehensive two-locus (*COI* + *18S rRNA*) molecular dataset with 43 Piscicolidae species (Table 1). Additionally, sequences of two Ozobranchidae species were collected as outgroup. Each partition was aligned separately using the MUSCLE algorithm of MEGA7^26^. The *18S rRNA* alignment was processed with GBlocks v0.91b^27^ using a set of available options for less stringent selection to eliminate hypervariable flanking and poorly aligned regions from the alignment (in summary, 74% of the initial alignment were excluded). The *COI* (665 bp) and *18S rRNA* (1698 bp) alignments were joined to a combined alignment using FaBox v1.5^28^. Divergence time was estimated using BEAST v1.10.4^29^. The best-fit evolutionary model HKY + G + I was applied to each partition. To dating the phylogeny, we used an external mean molecular rate for the *COI* (6.25 × 10^−9^ subst./site/year) and *18S rRNA* (1.99 × 10^−10^ subst./site/year) genes that were obtained based on a comprehensive fossil-calibrated phylogeny of the Hirudinea^2^. A lognormal relaxed clock algorithm and the Yule speciation process were applied as the tree priors. Two independent runs, each with 25,000,000 generations (sampling every 5000 cycles) were performed at the San Diego Supercomputer Center (SDSC, University of California, San Diego, USA) through the CIPRES Science Gateway^30^. The resulting log files were checked for convergence of the MCMC chains with Tracer v1.7^31^. The ESS values for all parameters were recorded > 200. The sets of time-calibrated trees obtained from the two runs were joined through LogCombiner v1.10.4^29^ with 10% burn-in. The maximum clade credibility tree was constructed based on 9000 binary time-calibrated trees using TreeAnnotator v1.10.4^29^.

Ancestral trait modeling was calculated using Bayesian Binary MCMC algorithm implemented in RASP v3.2^32^ based on the set of 9000 time-calibrated binary trees and the user-specified maximum clade credibility tree obtained in the previous BEAST analyses (see above). We coded three possible types of environmental preferences of leech species as follows: (a) freshwater, (b) marine, and (ab) euryhaline. The analysis was run with the following settings: 500,000 generations, sampling every 100th generation, 10 MCMC chains with temperature = 0.1 and 10% burn-in. Null distribution was not allowed.

### Nomenclatural acts

The electronic edition of this article conforms to the requirements of the amended International Code of Zoological Nomenclature (ICZN), and hence the new names contained herein are available under that Code from the electronic edition of this article. This published work and the nomenclatural acts it contains have been registered in ZooBank (https://zoobank.org), the online registration system for the ICZN. The LSID for this publication is as follows: https://zoobank.org/urn:lsid:zoobank.org:pub:33993F3F-FFD1-4688-8219-3F6D91D81A05. The electronic edition of this paper was published in a journal with an ISSN, and has been archived and is available from PubMed Central.

## Data Availability

The type series of the new species is available in the RMBH—Russian Museum of Biodiversity Hotspots, Federal Center for Integrated Arctic Research of the Ural Branch of the Russian Academy of Sciences, Arkhangelsk, Russia. The sequences used in this study are presented in GenBank.
